# A Practical Sensor-Based Methodology for the Quantitative Assessment and Classification of Chronic Non Specific Low Back Patients (NSLBP) in Clinical Settings

**DOI:** 10.3390/s20102902

**Published:** 2020-05-20

**Authors:** Mehrdad Davoudi, Seyyed Mohammadreza Shokouhyan, Mohsen Abedi, Narges Meftahi, Atefeh Rahimi, Ehsan Rashedi, Maryam Hoviattalab, Roya Narimani, Mohamad Parnianpour, Kinda Khalaf

**Affiliations:** 1 Department of Mechanical Engineering, Sharif University of Technology, Tehran 1136511155, Iran; mehrdaddavoodi15@gmail.com (M.D.); M.shokouhyan@gmail.com (S.M.S.); hoviat@sharif.edu (M.H.); narimani@sharif.edu (R.N.); parnianpour@sharif.edu (M.P.); 2 Physiotherapy Research Center, School of Rehabilitation, Shahid Beheshti University of Medical Sciences, Tehran 1616913111, Iran; mohsenabedi110@sbmu.ac.ir; 3 Physical Therapy Department, School of Rehabilitation Sciences, Shiraz University of Medical Sciences, Shiraz 7194733669, Iran; meftahin@sums.ac.ir; 4 Rehabilitation Sciences Research Center, Shiraz University of Medical Sciences, Shiraz 7194733669, Iran; 5 Department of Physical Therapy, University of Social Welfare and Rehabilitation Sciences, Tehran 1985713871, Iran; at.rahimi@uswr.ac.ir; 6 Department of Industrial and Systems Engineering, Rochester Institute of Technology, Rochester, NY 14623, USA; exreie@rit.edu; 7 Department of Biomedical Engineering and Health Engineering Innovation Center, Khalifa University of Science and Technology, P.O. Box 127788 Abu Dhabi, UAE

**Keywords:** wearable inertial sensor, low back pain (LBP) classification, clinical settings, quantitative screening

## Abstract

The successful clinical application of patient-specific personalized medicine for the management of low back patients remains elusive. This study aimed to classify chronic nonspecific low back pain (NSLBP) patients using our previously developed and validated wearable inertial sensor (SHARIF-HMIS) for the assessment of trunk kinematic parameters. One hundred NSLBP patients consented to perform repetitive flexural movements in five different planes of motion (PLM): 0° in the sagittal plane, as well as 15° and 30° lateral rotation to the right and left, respectively. They were divided into three subgroups based on the STarT Back Screening Tool. The sensor was placed on the trunk of each patient. An ANOVA mixed model was conducted on the maximum and average angular velocity, linear acceleration and maximum jerk, respectively. The effect of the three-way interaction of Subgroup by direction by PLM on the mean trunk acceleration was significant. Subgrouping by STarT had no main effect on the kinematic indices in the sagittal plane, although significant effects were observed in the asymmetric directions. A significant difference was also identified during pre-rotation in the transverse plane, where the velocity and acceleration decreased while the jerk increased with increasing asymmetry. The acceleration during trunk flexion was significantly higher than that during extension, in contrast to the velocity, which was higher in extension. A Linear Discriminant Analysis, utilized for classification purposes, demonstrated that 51% of the total performance classifying the three STarT subgroups (65% for high risk) occurred at a position of 15° of rotation to the right during extension. Greater discrimination (67%) was obtained in the classification of the high risk vs. low-medium risk. This study provided a smart “sensor-based” practical methodology for quantitatively assessing and classifying NSLBP patients in clinical settings. The outcomes may also be utilized by leveraging cost-effective inertial sensors, already available in today’s smartphones, as objective tools for various health applications towards personalized precision medicine.

## 1. Introduction

In a world that is rapidly embracing precision medicine and patient-centric state-of-the-art tools and technologies designed for healthcare, the quantitative classification of non-specific low back pain (NSLBP) and hence effective personalized treatment, remain elusive in most clinical settings. Indeed, while, several NSLBP classification systems were developed in the last few decades, their incompleteness and/or complexity, have led to the use of simpler tools. These include observational assessment, surveys and questionnaires, such as the STarT Back Screening Tool (STarT) [1]. This tool classifies NSLBP patients into three specific subgroups—low risk, medium risk and high risk based on 9 questions spanning educational therapy, physical therapy, as well as physical therapy combined with cognitive behavioral therapy. Risk is defined as the risk of developing persistent disabling symptoms [1]. Simplicity, ease of application and attention to psychosocial factors are key reasons that have made this multi-language questionnaire a valuable screening tool in clinical practice [2,3,4]. Abedi et al. found an approximately 80% correlation between the translated STarT questionnaire and other screening questionnaires, including the Roland Morris Disability Questionnaire (RMDQ), Tampa Scale for Kinesiophobia (TSK), Coping Strategies Questionnaire (CSQ) and Hospital Anxiety and Depression Scale (HADS) [4]. The McKenzie [5] method has also demonstrated practicality in clinical settings. This approach divides patients into three mechanical subgroups (derangement, dysfunction or postural syndrome) and applies appropriate exercises [5].

Marras et al. [6,7] used the Quebec Task Force Questionnaire to categorize patients with low back disorders (LBDs), in conjunction with the Lumbar Motion Monitor system (LMM) developed by these authors. They aimed to demonstrate that kinematic parameters, particularly angular velocity and acceleration, can be effectively used for patient classification and distinction between healthy individuals and low back pain (LBP) patients. Using a three-dimensional electromagnetic tracking system, Barrett et al. [8] indicated that the plane and direction of movement could influence the kinematic variables of low back patients. Sheeran et al. [9] also used a three-dimensional kinematics motion capture system to assess spinal and pelvic kinematics during sitting and standing activities in healthy and LBP patients. O’Sullivan et al. [10] introduced a multi-dimensional classification system (MDCS) to stratify patients based on movement and pain-based subsets (flexion pattern (FP), active extension pattern (AEP) and passive extension pattern (PEP)). Based on the novel Cardiff Classifier method, they were able to discriminate between different LBP subgroups with an accuracy of 96.8%, 87.7% and 70.27% for FP from PEP, FP from AEP and AEP from PEP subtypes, respectively. The MDCS system, however, is based on the range of motion and pain score of individuals during task performance without consideration of any psychosocial aspects. The need for the presence of a trained expert for patient evaluation is another limitation of this method, especially during remote evaluation. In general, despite their success in research settings, marker-based motion capture systems have limited accessibility and functionality for clinical applications, emphasizing the need for portable systems with sufficient accuracy and reliability.

Wearable inertial sensors have recently emerged as effective tools for objective quantitative LBP assessment due to their low cost, accuracy and portability [11,12,13]. Inertial Measurement Units (IMUs) are a combination of accelerometers, gyroscopes and/or magnetometers [14]. An accelerometer measures the linear acceleration of the sensor’s 3D local coordinate system [15], while an orthogonal gyroscope measures the angular velocity with respect to the sensor-embedded reference frame [16]. Magnetometers measure the direction and magnitude of the earth’s magnetic field [16]. Adding a magnetometer reduces orientation errors as calculated by the accelerometer and gyroscope [17]. Data fusion algorithms, such as Kalman or Complimentary filters, are typically utilized to estimate 3D orientations, based on the combined signals obtained from the accelerometer, gyroscope and magnetometer [14]. Navigation errors, which may occur during high acceleration movements and magnetic disturbance, should be accounted for [14,18]. Commercially available IMUs incorporate approaches such as a rotation matrix or Euler angle to quantify the captured kinematics in a global fixed frame [19]. Ashouri et al. [20] used a Support Vector Machine classifier to discriminate between LBP patients and healthy controls, by capturing the angular velocity and acceleration vectors during flexion-extension movements using two inertial sensors placed on the trunk and Pelvis. They concluded that simple flexion-extension movement in the sagittal plane, measured with only one sensor placed on the trunk, could distinguish healthy individuals from LBP patients with an accuracy of 96%, sensitivity of 100% and specificity of 92%. Esfahani et al. [21,22] developed and validated the inertial sensors used in this study (SHARIF-HMIS). Repeatability and reliability of these sensors were investigated by Abedi et al. [23]. Gorganbeik et al. [24] also assessed knee kinematics during landing tasks using the SHARIF-HMIS.

The main objective of the current study is to present a “sensor-based” practical methodology, that combines our previously validated inertial sensor with the STarT clinical screening tool. The methodology will be applied for quantitatively assessing and classifying NSLBP patients in clinical settings, towards tailored personalized interventions for LBP patients, as well the ability to objectively monitor and evaluate the efficacy of these interventions.

## 2. Materials and Methods

### 2.1. Participants

One hundred males with NSLBP were recruited from the physiotherapy clinic of Milad Hospital in Tehran. The inclusion criteria comprised of male patients aged between 20 and 50 years, with a history of LBP in the last year lasting at least three months and without any history of spinal deformities or surgery. The pain intensity of the patients needed to be less than 5 based on the Visual Analogue Scale (VAS) at the time of the test. Patients were excluded if they were unwilling or unable to participate. Prior to performing the experiment, the consent form (approved by Institutional Review Board (IRB) of Shahid Beheshti University of Medical Sciences, Tehran, IR) was reviewed and signed by all participants.

The demographic characteristics of the patients in each subgroup are presented in Table 1. There were no significant differences among the three subgroups in terms of age, height, weight and pain level based on VAS (Table 1).

### 2.2. Experimental Design

An inertial sensor (9DOF SHARIF-HMIS [21]), enclosing a 3-axis accelerometer (Analog Devices Inc., Norwood, MA, USA), a 3-axis gyroscope (InvenSense Inc., San Jose, CA, USA) and a 3-axis magnetometer (Honeywell Inc., Morris Plains, NJ, USA) with a 100 Hz frequency, was mounted vertically on the Xiphoid process of all participants. The captured data was transmitted to a PC-based data logger using an AVR microcontroller (Atmel Corp., San Jose, CA, USA). The technical information on the SHARIF-HMIS system is detailed in Esfahani et al. [21,22].

**Figure 1:**
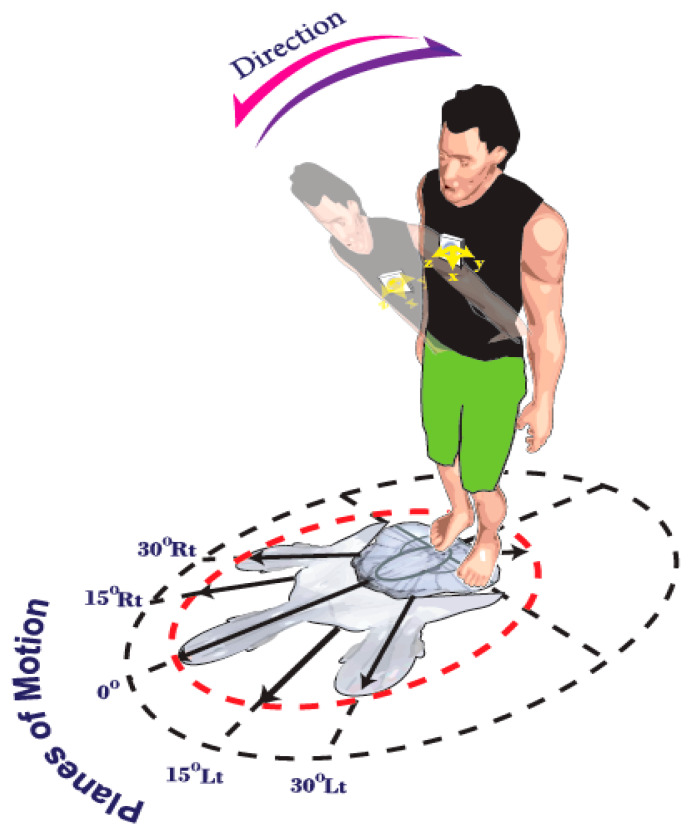
Schematic diagram of trunk repetitive motion spanning 5 planes of motion in two directions (flexion-extension) as depicted by Marras et al. [7].

All patients were asked to complete the STarT questionnaire before the experimental test. They wore a stretchable shirt, on which the inertial sensor was placed, in a manner minimizing relative movement. The participants were then asked to perform trunk flexion-extension movements in multiple planes to familiarize themselves with the setup. The experimental methodology was designed according to Marras et al. [6,7]. The patients performed flexion-extension trunk movements in five different planes of motion (PLMs) (sagittal plane (0°), 15° and 30° of right and left rotation, respectively) for a duration of 14 s, using their maximum comfortable and pain free speed. The angular velocity and acceleration data were directly acquired by the gyroscope and accelerometer. Figure 1 depicts the defined experimental planes of motion, as well as, the direction of the trunk’s flexion/extension. Due to the position of the sensor (vertically placed), the angular rate was dominant along the *Y*-axis of the gyroscope during flexion-extension and hence the data for this axis (angular velocity and linear acceleration) were used for subsequent analysis.

To avoid moving off plane, especially during asymmetrical movements, 5 lines were clearly marked on the ground at 5 different positions, ensuring that the participants restricted their movement to the intended plane (Figure 1). Here, asymmetry was defined as the amount of trunk twist (i.e., rotation in the transverse plane around the proximal-distal axis). The participants were asked not to use their ankle, knee or hip joints during twisting. The order of the planes of motion for each patient during the test was randomized. To avoid fatigue, the patients had a rest period for two minutes after each testing stage [23].

### 2.3. Data Processing

All data were analyzed using codes written in MATLAB (Mathworks, Inc., Natick, MA, USA). A 4th order Butterworth low pass filter, with a 10 Hz cutoff frequency, was applied to the raw data. A Fast Fourier Transform (FFT) was also utilized to compute the frequency components of the signals [25]. Although the duration of movement was the same for all patients (14 s), the number of cycles differed among them, since they were asked to do flexion-extension movements as fast as they comfortably could. The method employed by Ashouri et al. [20] was also used here to define the onset of movement. In each identified cycle, five kinematic variables, including maximum and average angular velocity, linear acceleration and maximum jerk (the second derivative of the angular velocity) were obtained from the sensors’ filtered data in both flexion and extension directions.

### 2.4. Statistical Analysis

One-way ANOVA and Kruskal-Wallis tests were used to compare the demographic data and VAS among the three groups. A mixed ANOVA model was applied on the dependent variables of velocity, acceleration and jerk, to investigate the kinematic differences among the 3 subgroups. The between-subjects factors included the three different subgroups (low risk, moderate risk and high risk NSLBP), while the within-subjects factors consisted of the direction (flexion and extension) and planes of motion (0° rotation, 15° right rotation, 15° left rotation, 30° right rotation, 30° left rotation). All statistical analyses were performed using IBM^®^ SPSS, version 20 (IBM Corporation, Armonk, NY). The level of significance for all tests was set at p < 0.05. Normality was tested using a Kolmogorov-Smirnov test.

### 2.5. Linear Discriminant Analysis

In this study, the Linear Discriminant Analysis (LDA) method was used to classify patients based on kinematic data [26]. Using IBM^®^ SPSS software (IBM SPSS Statistics for Windows, Version 25.0. Armonk, NY: IBM Corp), the purpose of the LDA was to determine the appropriate conditions in which subgroup separation is performed. All conditions (10 permutations: 5 PLMs × 2 directions) were evaluated for each of the 5 measured kinematic variables (Section 2.3), in order to discriminate among the three subgroups. The 15 degrees right axial pre-rotation position during extension was identified here as the condition with the highest discrimination based on kinematic behavior (see Table 6 in Section 3.2). Hereon, the 15° right axial pre-rotation during extension model will be labeled as Model 1.

In general, different methods can be used for analyzing data using LDA. Here, the feature selection stepwise method was selected. In this method, the appropriate variables to separate the groups can be identified by considering the remaining variables in the last step of the analysis. No other measures need to be included due to correlation with the selected variables. Upon examining the output of Model 1 (15° right axial pre-rotation during extension), Model 2 (extension in the planes of 15° right axial pre-rotation and 30° left axial pre-rotation) was developed to study the effect of adding conditions on the classifier’s performance. Using stepwise approach, only measures selected as the result of Model 1 were considered as suitable input variables for model 2. Section 3.2 provides further explanation.

Next, a discriminative analysis was performed to distinguish the high-risk subgroup from the low-moderate risk combination group, since this group is the most critical form diagnostic and prognostic perspectives. 32 subjects were randomly selected out of 68 from the low and moderate subgroups. The conditions in models 1 and 2 were fixed and the procedure was repeated 10 times to decrease random selection.

## 3. Results

### 3.1. Kinematic Variables

**Figure 2:**
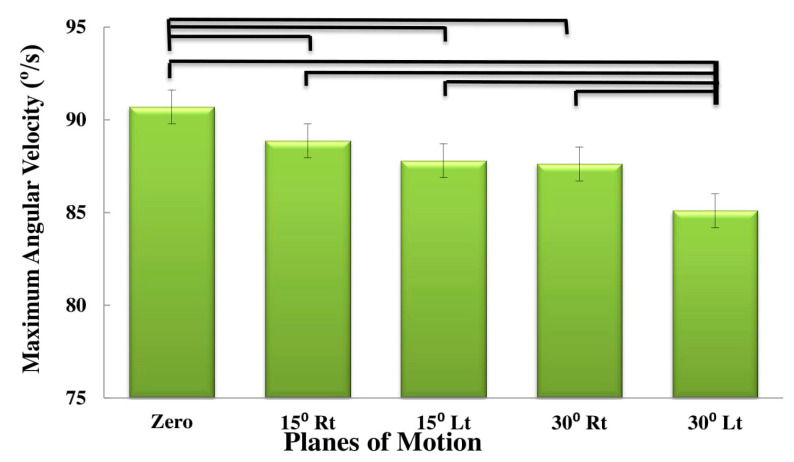
Main effects of planes of motion on maximum angular velocity.

**Figure 3:**
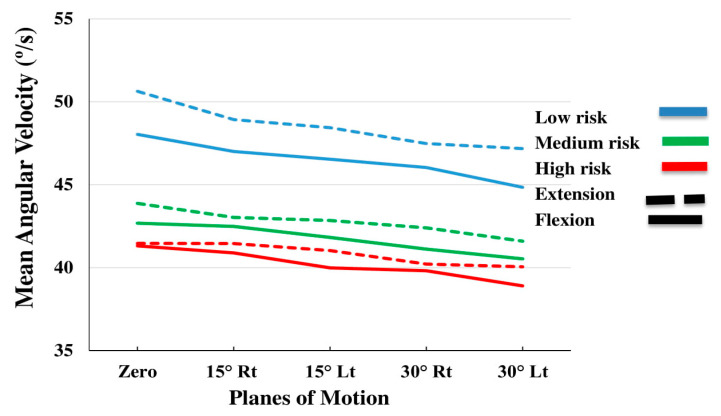
Interaction effects of Subgroup by planes of motion by direction on mean angular velocity.

**Figure 4:**
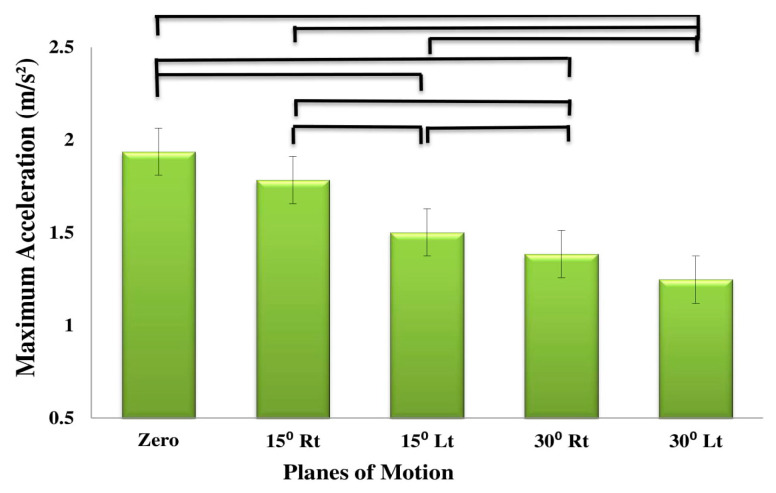
Main effects of planes of motion on maximum acceleration.

**Figure 5:**
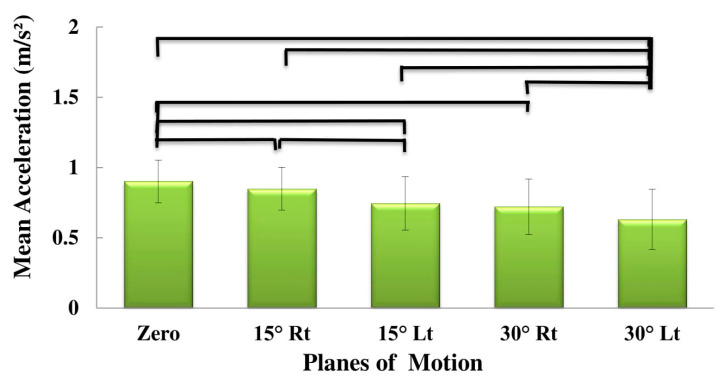
Main effects of planes of motion on the mean acceleration.

**Figure 6:**
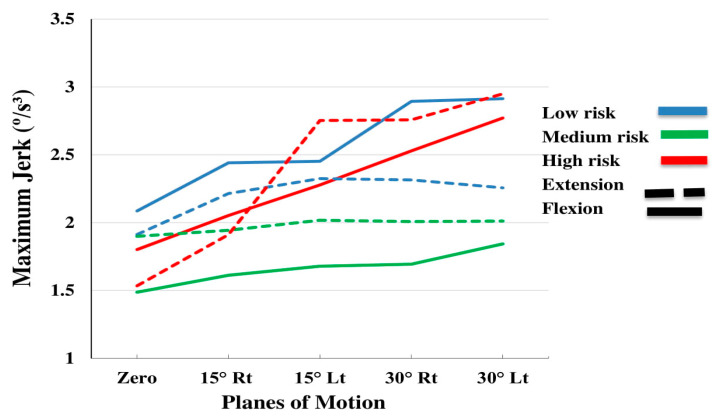
Interaction effects of Subgroup by planes of motion by direction on the maximum jerk.

Table 2 summarizes the ANOVA results of the effects of subgroup (low risk, medium risk, high risk), plane of motion (PLM) (0° rotation, 15° rotation to the right, 15° rotation to the left, 30° rotation to the right and 30° rotation to the left) and direction of motion (flexion and extension), on the kinematic variables (maximum and mean angular velocity, linear acceleration and maximum jerk). Table 3, Table 4 and Table 5 summarize the descriptive statistics. The effect of PLM was significant for all the variables (*p*-values < 0.01; Table 2). The effect of direction on the mean velocity and acceleration, as well as on the maximum jerk was also significant (*p*-values < 0.019; Table 2). As shown in Table 3, Table 4 and Table 5, the maximum velocity (Figure 2), mean velocity (Figure 3), maximum acceleration (Figure 4) and mean acceleration (Figure 5) variables revealed a decreasing trend with the increase in motion asymmetry (moving from 0° to 30°) in both flexion and extension directions, for each of the three subgroups. The velocity and acceleration of the trunk motion in the sagittal plane, as well as the 30° rotation to the left position, were significantly higher and lower, respectively, as compared to the other planes. On the other hand, the trend of the maximum jerk continued increasing (Figure 6). 

**Figure 7:**
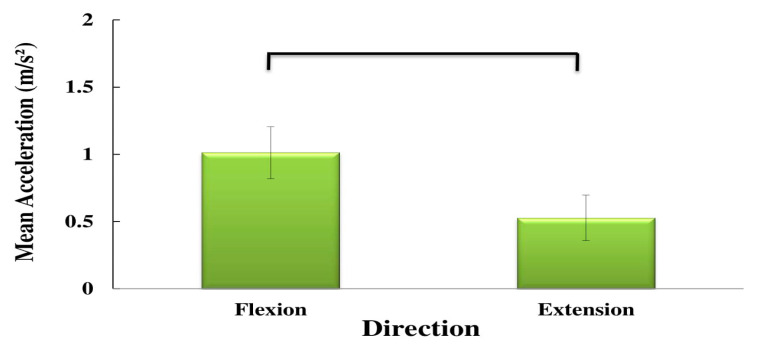
Main effects of direction on the mean acceleration.

The mean velocity values were significantly higher during extension (Figure 3), while the mean acceleration values were higher during flexion (Figure 7). The maximum jerk was higher for the high and medium risk subgroups (Figure 6). No significant interaction effects of the direction on the maximum velocity and acceleration were observed.

**Figure 8:**
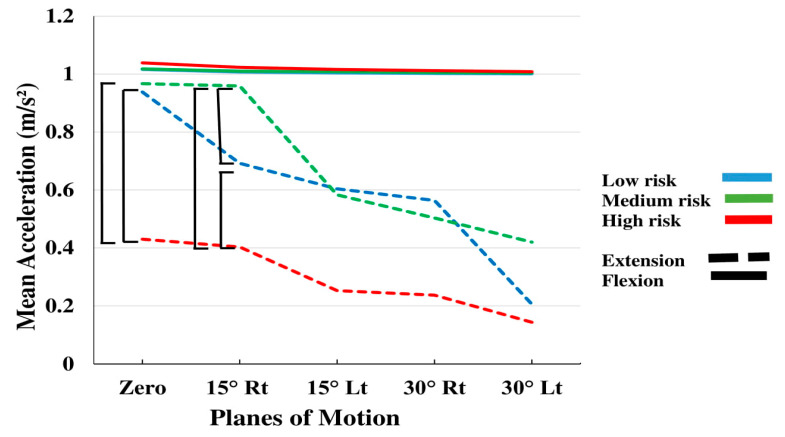
Interaction effects of Subgroup by planes of motion by direction on mean acceleration.

The effect of subgroup × PLM interaction on the mean acceleration approached significance (*p*-values = 0.07; Table 2), while the effect of the three-factor group interaction of Subgroup × PLM × direction was significant (*p*-values < 0.05; Table 2). The mean acceleration demonstrated more significant subgroup effect (*p*-values = 0.06; Table 2), as compared to other extracted kinematic trunk variables. In Figure 8, the influence of all three subgrouping factors, PLM and direction can be clearly seen on the mean acceleration values. While the values of the three subgroups were very close during flexion movements, the differences among them became more evident during extension. The high-risk group revealed the lowest acceleration values in planes of motion, especially at zero degrees, where this difference was significant. No clear difference was observed between the low risk and medium risk subgroups.

Post-hoc analysis revealed that at 15 degrees right axial pre-rotation during extension, there were significant differences among the three subgroups. Extension in the sagittal plane also demonstrated significant differences between the high risk and low-medium risk subgroups.

### 3.2. Discriminant Analysis

As previously mentioned, two models were evaluated using linear discriminant analysis (LDA). In Model 1 (15 degrees right axial pre-rotation during extension), LDA was used to select a specific condition based on the classification performance between 10 different scenarios (5 PLMs × 2 directions). A 100 × 5 matrix (100-rows (number of patients in the three Subgroups) and 5-columns (all the kinematic variables, including maximum and average velocity, maximum and average acceleration and maximum jerk)) was used as the input for each condition. As shown in Table 6, Model 1 revealed the highest discrimination performance among the three STarT. Subgroups.

Based on Model 1, more than 50% of the patients could be correctly classified into their subgroup (Table 7). This model, however, performed better for the high-risk group as compared to low-medium risk patients, providing more than 65% (21 correctly diagnosed out of 32 patients) correct diagnosis. Conversely, it was less successful in classifying medium risk patients (approximately 35%) and only partially diagnosed patients in the low risk group. Moreover, only mean acceleration variable remained in the last step of the analysis. The average acceleration was an effective measure in separating the three subgroups, while the other variables did not provide additional information in this model. Equations (1)–(3) demonstrate that the classification functions for each subgroup, −1.379, −0.901 and −2.881, respectively, are the coefficients of functions derived from the discriminant analysis.

(1) $$\mathrm{Low}=(-1.379\times Acc_{mean})+1.639$$

(2) $$\mathrm{Medium}=(-0.901\times Acc_{mean})+1.329$$

(3) $$\mathrm{High}=(-2.881\times Acc_{mean})+2.178.$$

Therefore, a new patient would be classified by calculating the total of all three classification functions (by summing the constant and products of the patient’s kinematic results (mean acceleration), as well as the coefficient, as shown in the above equations) and consequently would be assigned into the subgroup which yielded the highest score. For example, a patient with an average acceleration of 0.3, the calculated function scores would be 1.255, 1.059 and 1.313, respectively and hence the patient would be assigned to the high-risk subgroup. The performance of these functions in classifying patients is shown in Table 7. The results of Model 1 revealed weakness in the separation of the medium risk group. Based on Table 7, using the 30° rotation to the left in extension scenario, enhances the performance of discrimination. A second model was, therefore, developed to combine both the 15° rotation to the right and the 30° rotation to the left, in extension, under one model (Model 2).

The purpose of the second modeling attempt was not to specify the selected condition or measure but rather to understand how and by how much the addition of another condition (direction-plane) can improve the performance of the model. For demonstration, a similar measure selected from Model 1 (mean acceleration) was analyzed in Model 2, resulting in 100 rows (number of patients) and 2 columns (selected variables in two conditions). The use of the Model 2 added only add 4% to the overall performance. The separation status for the medium and low risk groups improved by approximately 9% and 5%, respectively, while the high-risk group separation performance decreased by 2%. Only the results in the allocated (true) table (Table 7) were reported to compare the performance of the two models.

As shown in Table 8, the discrimination performance increased by 66.9% and 65.7%, for Models 1 and 2, respectively, in terms of distinguishing the high-risk from the low-medium risk patients.

## 4. Discussion

The current study presented a novel methodology towards the practical clinical application of inertial sensors as a quantitative tool to classify NSLBP. Various relevant kinematic variables among three subgroups of patients (low, high and medium risk) with NSCLBP were measured and compared. The results can be summarized as follows—(1) Increasing asymmetry in the planes of motion, resulted in decreasing the acceleration and angular velocity, while increasing the jerk; (2) The angular velocity and jerk were higher during extension, while the acceleration was higher during flexion. (3) Trunk extension position of 15 degrees rotation to the right can potentially be used to identify LBP patients, especially in the high-risk group.

### 4.1. Planes of Motion Effect

The results demonstrated a decrease in the angular velocity and acceleration with increasing asymmetry. We hypothesize that during trunk flexion-extension in a symmetrical plane, the movement is mostly controlled by the large stabilizing spinal muscles. As the angle of motion changes from zero, either to the right or left, the contribution of the smaller external and internal abdominal muscles is increased to control the movement [27]. This shift from large stabilizer muscles to secondary smaller muscles may have caused a decrease in the velocity and acceleration. Control of asymmetric motion typically requires a high degree of muscle co-contraction. Increasing the number of the involved muscles, increases the time needed to control the activity of these muscles, which may lead to slower movement [28].

Ogon et al. [29] reported jerk as a natural part of relatively rapid movements. They suggested that changes in the strain rate of the viscoelastic biological lumbar tissues, as well as the axis of spine rotation, during flexion-extension may contribute to the jerk. Indeed, in the present study, the observed increase in jerk was associated with increasing asymmetry. It is possible that as the angular velocity and acceleration became more non uniform with increasing asymmetry, producing higher jerk values.

### 4.2. Direction Effect

The higher velocity observed during trunk extension, as compared to flexion during asymmetric movement, may be attributed to the stronger extensor muscles (vs. flexor muscles). Although Kim et al. [30] proposed a flexion-relaxation phenomenon for extensor muscles in healthy individuals, they reported that this phenomenon is not replicated in LBP patients. The extensor muscles of these patients are constantly active during flexion, which may impair flexion. This may be considered as a potential reason for the decreased flexion velocity seen in the three subgroups of LBP patients in the present study.

The results also revealed less acceleration during extension and yet greater jerk in the same direction, which seems to indicate control challenges during extension in these patients, especially in the high-risk group, who had the least acceleration (Figure 8). Various factors, such as fear of pain and a history of pain during extension, in addition to weak lumbar extension muscles can affect the patient’s movement. Moreover, structural, neurophysiological and behavioral changes in the lumbar spine may lead to a decrease in the deep sense and awareness of the patient’s lumbar movements, especially during extension [31].

### 4.3. Subgrouping Effect

Using inertial sensors for the assessment of the trunk kinematic indices, Ashouri et al. [20] were able to classify the participants into 2 subgroups of healthy and LBP patients. They found that an experimental protocol, based on a single sensor placed on the chest during simple flexion-extension tasks in the sagittal plane, can identify LBP patients with an accuracy of 96% and a sensitivity of 100%. In our study, all subjects were NSLBP. They had already been divided into three subgroups of low, medium and high risk (based on the clinical classification). Our results indicate that the kinematic indices were not always significantly different between the subgroups in flexion (Figure 8). The mean acceleration was significantly affected by subgrouping, while the interaction of Subgroup by PLM approached significance. In addition the three-way interaction of Subgroup by PLM by direction was significant.

One of the reasons that subgrouping did not have a significant effect on some of the kinematic variables, may be the bio-psycho-social STarT questionnaire, which despite its simplicity and clinical popularity, remains a subjective tool that is not designed to account for kinematic parameters. In addition, in this study, all patients were at approximately similar pain level (VAS < 5) during testing. As can be seen in Figure 8 and the results of the LDA analysis, identifying high-risk individuals can well be made based on their kinematic status.

### 4.4. Linear Discriminant Analysis

The results of the discriminant analysis in the current study suggest that by examining the trunk kinematic profiles of a patient in defined conditions, the health care provider may be able to better assess the level of the patient’s impairment, especially if it is high risk, towards prescribing more effective treatment/rehabilitation modalities. Evidence-based and theoretical considerations indicate that correctly identifying high-risk patients is critical and yet often neglected in clinical practice, despite the negative consequences of missing the chance to provide timely cognitive therapy, in addition to physiotherapy.

This study aimed to introduce a specific methodology/protocol for the discrimination of LBP patients, based on the risk/level of LBP, by using inertial sensors. Contrary to the study of Sheeran et al. [9], which requires a well-equipped laboratory space, the portable wearable inertial sensors and protocol presented here, can be easily applied in clinical settings. Furthermore, using the STarT questionnaire, unlike other subgrouping methods of LBP patients such as O’Sullivan [10], does not require any specific expertise and can be easily integrated in modern clinical practice. Moreover, flexion/extension tasks are among the simplest possible movements that a patient can safely perform under supervision. As recommended by Ashouri et al. [20], only one sensor is needed, which makes the application inexpensive and convenient.

This study revealed a major difference in extension between the LBP subgroups, particularly for the acceleration variable. However, contrary to the results of Ashouri et al. [20], who distinguished LBP patients from healthy subjects in a zero-degree symmetrical plane with an accuracy of 96%, our study indicated that a 15-degree of asymmetry was needed to better classify the three subgroups. Based on the identified differences, three simple equations were formulated for quantification. By inserting the acceleration value in these equations, the subgroup could be predicted, with some error, especially the high-risk group. The best discrimination percentage obtained in this study is well within the range described in the literature using inertial sensors in clinical applications (~70%) [32,33,34,35]. Brandt et al. [36] used LDA on trunk angular data for discriminating people who performed high-risk and low risk lifting tasks. They were able to separate these individuals with 65% accuracy, which was enhanced to 75% by adding electromyography data.

In summary, the following are some notable points of discussion:

- All participants in the present study were patients with LBP. In the previous study, using the same inertial sensors, the discrimination accuracy between healthy individuals and LBP patients was approximately 96% [20]. Since the current investigation only included patients, the trunk kinematic variability among these three subgroups may be less than those between healthy participants and patients. Further research is needed for validation.
- According to the STarT questionnaire, a patient with scores of 3 and 4 is classified as low risk and medium risk, respectively. Many patients, however, may be on the boundary between these two subgroups. Classified as “cusp patients,” these individuals are currently separated by only one affirmative answer on the questionnaire. This process may clearly increase the classification error, potentially explaining the low achieved discrimination percentage between the low and medium risk subgroups observed here.
- Sheeran et al. [9] revealed good classification results among the defined subtypes of LBP patients. However, they did not evaluate the pain score of these patients. Significant differences in the pain score can affect the separation results. In the present study, the pain score was not significantly different among the subgroups of patients during testing. Subgrouping in the O’Sullivan approach was performed by an expert clinician through examining the lumbar range of motion during active and passive flexion/extension, as long as the patient feels pain [10]. Therefore, it can be postulated that pain is an important factor for distinguishing patients, as it directly affects classification and should not be eliminated from assessment protocols.

In a companion study, Abdollahi et al. (published in preprint) [37], used inertial sensors for the classification of subgroups of LBP patients. They could distinguish high risk subgroups from low and medium risk patients with a 76% accuracy by applying the SVM method on trunk movement patterns. Using a simpler approach, such as LDA applied in the present study, makes it easier for non-engineers, especially therapists and clinicians, to better understand the biomechanical factors in play and kinematic variability among different subgroups. The simplistic quantitative approach adopted in this study may bridge the wide existing gap between biomechanists and health care providers to assist patients more effectively.

Our sample size was 100 patients with approximately 33 participants in each subgroup. Ashouri et al. [20] studied 28 patients and 24 healthy individuals, while Sheeran et al. study assessed 87 patients in total [9]. The sample sizes, including three subgroups with flexion pattern, active extension pattern and passive extension pattern, were 49, 23 and 14 LBP patients, respectively. Since both previous studies reported higher classification percentage, the effect of sample size on subgrouping performance should be examined in future studies. Esfehani et al. [38] used a method known as Bayes [39] to investigate the maximum possible accuracy of classification using wearable sensors. This method may be utilized to identify the best possible accuracy that can be achieved using the data collected in the present study, independent of any specific classification method.

## 5. Limitations

LDA was the only method used in this study to discriminate among the three LBP patient subgroups. With the help of machine learning and nonlinear dynamic methods, more improved discriminant analyses tools can be exploited in future studies towards better results of accuracy and sensitivity. By analyzing the temporal patterns of the variables measured by the sensor, instead of standard indicators, such as maximum and mean, the behavior of patients in the three subgroups can be compared in real time during dynamic movement. Another limitation of this study is that due to the hospital’s logistics, only male participants were recruited and hence the kinematic profiles of females were not examined. Moreover, all the participants were right-handed, which may have influenced the results, as it is usually easier to move to the dominant side. Although the sensor was tightly attached, some error may have resulted from sensor placement. An adaptive Kalman filter [40] was implemented in the SHARIF-HMIS system in order to reduce the error based on high-acceleration movement and to separate the linear acceleration variable from the gravity component [21,22]. In the current study, due to the lack of access to these programs and the limitations in the original clinical study that precluded the inclusion of added static postures needed for proper calibration [12,21,22], the output of the IMU sensors was directly used without adaptation. This also prevented the estimation of trunk angular position, as well as the compensation of the gravitational acceleration in the output of linear accelerometers. We have partially compensated for this shortcoming by dividing the repetitive movements into four phases of motion—acceleration/deceleration phases of trunk flexion and extension, respectively. Limited ankle movement or hip flexion was also unavoidable while performing the various movements. Furthermore, in the current study, the features were extracted only from the signals obtained in the *Y* axis of the sensor’s local reference frame due to the domination of the angular velocity in this direction during flexion/extension as previously mentioned. The signals captured by the sensor in the X and Z directions were not included in our analysis. Locally measured *Y*-axis-signals provided enough information on patients’ trunk kinematics for this study. Future work, however, should study other available signals.

## 6. Conclusions

This work described a methodology towards the application of wearable inertial sensors as a quantitative tool to classify NSLBP in clinical settings. The results identified a particular trunk position (extension at 15° asymmetry to the right) as an appropriate benchmark for risk stratification, particularly for high risk NSLBP patients. Further work is needed to validate and expand this model, exploring other nonlinear dynamic tools, as well as machine learning. The framework described here may be implemented by leveraging cost-effective inertial sensors, already available in today’s smartphones, as objective tools for various health applications, including NSLBP quantitative kinematic assessment and continuous monitoring in the pursuit of personalized precision medicine.

## Figures and Tables

**Table 1:** Demographic characteristics (mean ± SD) of NSLBP patients classified by STarT.

NSLBP (100 Males)			
Variables	Low Risk (*n* = 33)	Medium Risk (*n* = 35)	High Risk (*n* = 32)
**Age (year)**	46.1 ± 3.2	44.8 ± 4.4	44.3 ± 3.4
**Height (cm)**	172.1 ± 7.1	173.4 ± 6.7	171.3 ± 7.6
**Weight (kg)**	79.2 ± 13.8	81.8 ± 11.2	79.1 ± 12.4
**VAS**	4.1 ± 0.4	4.4 ± 0.2	4.2 ± 0.3

**Table 2:** Summary of ANOVA results (*p*-values) of the effects of subgroup (low risk, medium risk, high risk), planes of motion (0° rotation, 15° rotation to the right, 15° rotation to the left, 30° rotation to the right and 30° rotation to the left) and direction (flexion and extension) on the maximum and mean angular velocity and linear acceleration and maximum jerk.

Effects of ANOVA	Angular Velocity (°/s)		Linear Acceleration (m/s^2^)		Jerk (°/s^3^)
	Max	Mean	Max	Mean	Max
Subgroup	0.19	0.25	0.69	0.06	0.39
Planes of Motion (PLM)	0.01 *	<0.001 *	<0.001*	<0.001 *	<0.001 *
Flexion-Extension (Direction)	0.13	<0.001 *	0.77	0.003 *	0.019 *
Subgroup × PLM	0.39	0.24	0.19	0.07	0.47
Subgroup × Direction	0.45	0.89	0.09	0.27	0.32
Direction× PLM	0.57	0.32	0.23	0.33	0.54
Subgroup × Direction × PLM	0.84	0.54	0.45	0.05 *	0.29

* Statistical significant (*p* < 0.05).    Approaching significant.

**Table 3:** Descriptive statistics (mean ± SD) of the mean and maximum angular velocity for the 3 subgroups of NSLBP (low risk, medium risk, high risk) in the 5 planes of motion (0° rotation, 15° rotation to the right, 15° rotation to the left, 30° rotation to the right and 30° rotation to the left) in 2 directions (flexion and extension).

Kinematic Variables		Angular Velocity (°/s)											
		Max						Mean					
Position		G1		G2		G3		G1		G2		G3	
		Mean	SD	Mean	SD	Mean	SD	Mean	SD	Mean	SD	Mean	SD
Flexion	Zero	104.23	6.74	84.91	6.54	80.67	6.84	48.03	3.60	42.67	3.502	41.32	3.66
	15° Rt	101.66	6.41	84.76	6.23	78.51	6.51	47.006	3.63	42.48	3.531	40.89	3.69
	15° Lt	99.932	6.35	84.55	6.17	76.55	6.45	46.535	3.66	41.82	3.556	39.91	3.71
	30° Rt	99.284	6.16	84.11	5.98	76.28	6.25	46.033	3.36	41.12	3.264	39.81	3.41
	30° Lt	93.577	6.16	83.99	5.99	75.28	6.26	44.841	3.44	40.52	3.343	38.99	3.49
Extension	Zero	101.7	5.74	86.47	5.57	86.14	5.83	50.632	3.21	43.87	3.119	41.46	3.26
	15° Rt	96.676	5.69	85.50	5.52	86.13	5.78	48.921	3.21	43.02	3.126	41.45	3.26
	15° Lt	96.227	5.66	83.43	5.53	86.10	5.75	48.438	3.27	42.84	3.175	41.02	3.32
	30° Rt	96.155	5.49	83.79	5.33	86.10	5.57	47.485	3.37	42.39	3.273	40.21	3.42
	30° Lt	90.01	5.48	81.70	5.32	86.00	5.57	47.174	3.41	41.59	3.311	40.04	3.46

Where: G1: subgroup1 (low risk), G2: subgroup 2 (medium risk), G3: subgroup 3 (high risk), Rt: right, Lt: left.

**Table 4:** Descriptive statistics (mean ± SD) of the mean and maximum linear acceleration for the 3 subgroups of NSLBP (low risk, moderate risk, high risk) in the 5 planes of motion (0° rotation, 15° rotation to the right, 15° rotation to the left, 30° rotation to the right and 30° rotation to the left) in 2 directions (flexion and extension).

Kinematic Variables		Linear Acceleration (m/s^2^)											
		Max						Mean					
Position		G1		G2		G3		G1		G2		G3	
		Mean	SD	Mean	SD	Mean	SD	Mean	SD	Mean	SD	Mean	SD
Flexion	Zero	1.836	0.2	1.97	0.194	1.988	0.203	1.016	0.155	1.018	0.151	1.039	0.158
	15° Rt	1.426	0.217	1.954	0.211	1.987	0.221	1.007	0.155	1.01	0.15	1.023	0.157
	15° Lt	1.083	0.263	1.87	0.255	1.878	0.267	1.004	0.195	1.008	0.189	1.016	0.198
	30° Rt	1.03	0.216	1.578	0.21	1.802	0.219	1.002	0.243	1.005	0.236	1.012	0.247
	30° Lt	1.027	0.279	1.436	0.271	1.49	0.283	1.001	0.221	1.004	0.214	1.008	0.224
Extension	Zero	1.814	0.202	1.975	0.196	2.04	0.205	0.938	0.15	0.967	0.145	0.43	0.152
	15° Rt	1.374	0.219	1.958	0.212	2.008	0.222	0.692	0.153	0.959	0.148	0.403	0.155
	15° Lt	1.144	0.262	1.033	0.255	2.003	0.266	0.604	0.186	0.583	0.181	0.253	0.189
	30° Rt	1.02	0.228	1.027	0.221	1.851	0.231	0.564	0.153	0.503	0.148	0.237	0.155
	30° Lt	1.007	0.2	1.002	0.29	1.52	0.303	0.206	0.211	0.42	0.205	0.144	0.214

Where: G1: subgroup1 (low risk), G2: subgroup 2 (medium risk), G3: subgroup 3 (high risk), Rt: right, Lt: left.

**Table 5:** Descriptive statistics (mean ± SD) of the maximum jerk for the 3 subgroups of NSLBP (low risk, moderate risk, high risk) in the 5 planes of motion (0° rotation, 15° rotation to the right, 15° rotation to the left, 30° rotation to the right and 30° rotation to the left) in 2 directions (flexion and extension).

Kinematic Variables		Jerk (°/s^3^)					
		Max					
Position		G1		G2		G3	
		Mean	SD	Mean	SD	Mean	SD
Flexion	Zero	1.836	0.2	1.97	0.194	1.988	0.203
	15° Rt	1.426	0.217	1.954	0.211	1.987	0.221
	15° Lt	1.083	0.263	1.87	0.255	1.878	0.267
	30° Rt	1.03	0.216	1.578	0.21	1.802	0.219
	30° Lt	1.027	0.279	1.436	0.271	1.49	0.283
Extension	Zero	1.814	0.202	1.975	0.196	2.04	0.205
	15° Rt	1.374	0.219	1.958	0.212	2.008	0.222
	15° Lt	1.144	0.262	1.033	0.255	2.003	0.266
	30° Rt	1.02	0.228	1.027	0.221	1.851	0.231
	30° Lt	1.007	0.2	1.002	0.29	1.52	0.303

Where: G1: subgroup1 (low risk), G2: subgroup 2 (medium risk), G3: subgroup 3 (high risk), Rt: right, Lt: left.

**Table 6:** The performance of linear discriminant analysis (LDA) in 10 different conditions of motion for 3 subgroups of NSLBP.

**Condition**	0°_F	0°_E	15°_Rt_F	**15**°**_Rt_E**	15°_Lt_F	15°_Lt_E	30°_Rt_F	30°_Rt_E	30°_Lt_F	30°_Lt_E
**Performance (%)**	30	45	36	**51**	37	46	32	42	31	48

Abbreviation: F: flexion, E: extension, Rt: rotation to right, Lt: rotation to left.

**Table 7:** The performance of LDA in classifying patients into each subgroup (low, medium and high risk) by 2 different models (Model1: data related to movement of patients in extension in a plane of 15° rotation to the right, Model2: extension in planes of 15° rotation to the right and 30° rotation to the left).

Subgroup into Which Patient Classified by	Allocated (True) Subgroup by STarT					
	Models					
	1			2		
	Low	Medium	High	Low	Medium	High
Classification matrix						
Low	17	11	5	19	8	6
Medium	14	13	8	13	16	6
High	4	7	21	8	4	20
Number (%) correct by group	17/33 (51.5)	13/35 (37.1)	21/32 (65.6)	19/33 (57.5)	16/35 (45.7)	20/32 (62.5)
Total number (%) percent correct	51/100 (51)			55/100 (55)		

The first model’s performance was 51% (51 patients were correctly classified among a total of 100 patients). Amongst 33 patients in the low risk subgroup in model 1, 17 subjects were correctly classified, meanwhile 11 and 5 patients incorrectly classified into medium and high-risk subgroups respectively.

**Table 8:** The performance (%) of LDA in classifying the high-risk subgroup from low-moderate risk (mean ± SD), by 2 different models. Model1: data related to movement of patients in extension in a plane of 15° rotation to the right. Model2: data related to movement of patients in extension in planes of 15° rotation to the right and 30° rotation to the left.

	Model 1	Model 2
High vs. Low-Moderate risk	66.9 ± 2.1	65.7 ± 3.3

Discrimination analysis for high risk vs. low-moderate risk was repeated 10 times, the mean ± standard deviation is shown in the table.
